# A key role for MAM in mediating mitochondrial dysfunction in Alzheimer disease

**DOI:** 10.1038/s41419-017-0215-0

**Published:** 2018-02-28

**Authors:** Estela Area-Gomez, Ad de Groof, Eduardo Bonilla, Jorge Montesinos, Kurenai Tanji, Istvan Boldogh, Liza Pon, Eric A. Schon

**Affiliations:** 1 Department of Neurology, Columbia University, New York, NY USA; 2 Department of Pathology and Cell Biology, Columbia University, New York, NY USA; 3 Department of Genetics and Development, Columbia University, New York, NY USA; 4 Present Address: Intervet International bv., Wim de Körverstraat 35, 5830AA Boxmeer, The Netherlands

## Abstract

In the last few years, increased emphasis has been devoted to understanding the contribution of mitochondria-associated endoplasmic reticulum (ER) membranes (MAM) to human pathology in general, and neurodegenerative diseases in particular. A major reason for this is the central role that this subdomain of the ER plays in metabolic regulation and in mitochondrial biology. As such, aberrant MAM function may help explain the seemingly unrelated metabolic abnormalities often seen in neurodegeneration. In the specific case of Alzheimer disease (AD), besides perturbations in calcium and lipid homeostasis, there are numerous documented alterations in mitochondrial behavior and function, including reduced respiratory chain activity and oxidative phosphorylation, increased free radical production, and altered organellar morphology, dynamics, and positioning (especially perinuclear mitochondria). However, whether these alterations are primary events causative of the disease, or are secondary downstream events that are the result of some other, more fundamental problem, is still unclear. In support of the former possibility, we recently reported that C99, the C-terminal processing product of the amyloid precursor protein (APP) derived from its cleavage by β-secretase, is present in MAM, that its level is increased in AD, and that this increase reduces mitochondrial respiration, likely via a C99-induced alteration in cellular sphingolipid homeostasis. Thus, the metabolic disturbances seen in AD likely arise from increased ER-mitochondrial communication that is driven by an increase in the levels of C99 at the MAM.

## Facts

- Mitochondrial bioenergetic function is decreased in AD, but the reason for this decline is unknown.
- A “mitochondrial cascade hypothesis” has been put forward to explain AD pathogenesis.
- ER-mitochondrial communication and MAM function are increased significantly in AD.
- C99 is present in MAM, and accumulates above normal levels in AD cells and animal models.
- Increased C99-mediated MAM activity induces bioenergetic dysfunction in AD cells.

## Open questions

- How does C99 modulate MAM function in general and bioenergetic output in particular?
- What is the mechanism of mitochondrial dysfunction due to alterations in MAM behavior?
- How do these alterations occur in sporadic AD, in which APP processing is presumably normal?

## Introduction

Alzheimer disease (AD) is the most common adult neurodegenerative disorder^1^. Pathologically, it is characterized by progressive neuronal loss in the hippocampus and cortex, with the accumulation in the brain of extracellular neuritic plaques and intracellular neurofibrillary tangles. Prominent among the proteins deposited in the plaques is β-amyloid (Aβ), which is produced by cleavage of the amyloid precursor protein (APP) by presenilin-1 (PS1) and/or presenilin-2 (PS2), both of which are active components of the γ-secretase complex^2^. Notably, dominantly inherited mutations both in the presenilins and in APP are currently the only known causes of the familial form of AD (FAD), which has led to the most widely accepted hypothesis to explain the pathogenesis of AD, namely, the “amyloid cascade,” which proposes that deposition of Aβ in the brain is the precipitating pathological event in AD^3^. However, while the amyloid cascade hypothesis helps explain the development of the plaques and perhaps also the tangles, it sheds little light on the impact of other aspects of the disease, some of which occur years before the appearance of those plaques and tangles^4–6^. Those other aspects include altered metabolism of phospholipids and fatty acids^7,8^, increased levels of circulating cholesterol^9^, the deposition of lipid droplets within cells^10–12^, alterations in glucose levels^13^, aberrant calcium homeostasis^14^, increased ER stress^15^, and mitochondrial dysfunction^16,17^, the focus of our discussion here.

## Mitochondrial alterations in AD

In the last few decades, many reports have demonstrated the impairment of mitochondrial function in AD. Moreover, a number of lines of biochemical and cell biological evidence have been marshaled in support of a “mitochondrial cascade hypothesis” for the pathogenesis of AD, which proposes that mitochondrial alterations initiate the cascade of pathologies characteristic of the disease^18–25^. However, while this possibility is intriguing, it is currently unclear whether the impairment of mitochondrial function in AD^26–33^ is the cause, the consequence, or merely a “bystander effect” of the biochemical and morphological changes seen in AD^34,35^. While mitochondria are clearly altered in AD, we believe that the mitochondrial cascade hypothesis has a number of flaws, discussed in greater detail below, that have led us to the conclusion that mitochondrial dysfunction is an early disturbance in the pathogenesis of AD but is not the driver of the pathogenesis.

### Mitochondrial biochemical and dynamic alterations

As alluded to above, mitochondrial bioenergetic function is reduced in AD. Specifically, AD patients and animal models of AD exhibit reduced respiratory chain activity and lower ATP production^26,28,31,36–39^. Additionally, many reports have described a significant decrease in enzymes of the mitochondrial tricarboxylic acid cycle in AD patients^40–42^. Moreover, the levels of free radicals and reactive oxygen species, which are produced mainly by mitochondria, are elevated in AD cells^38,43–46^.

**Fig. 1:**
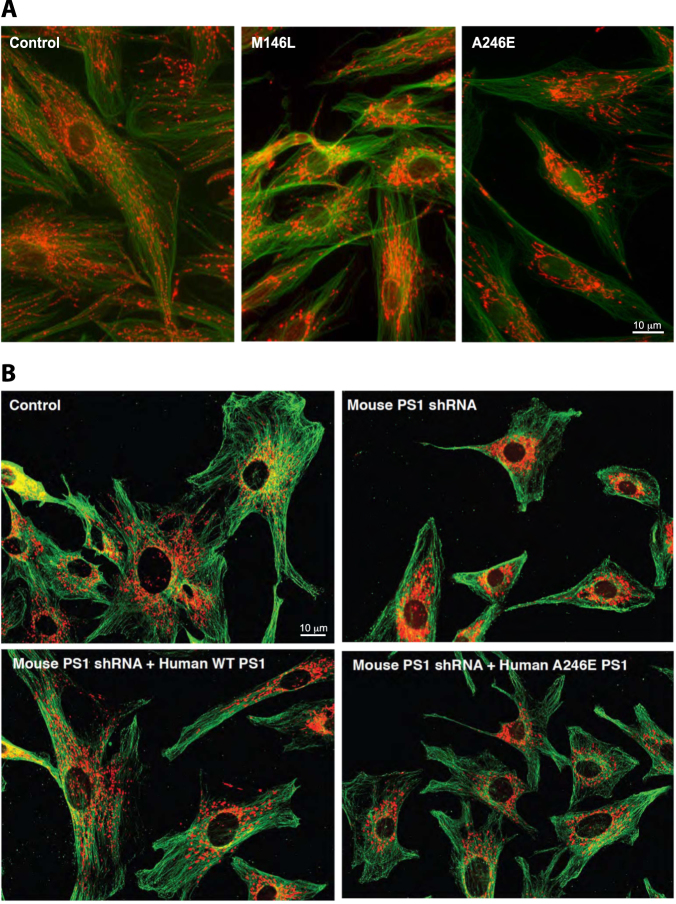
**a** Representative mitochondrial morphology in AD-mutant cells. Human fibroblasts were stained with MitoTracker (red) and anti-tubulin (green). Note the relatively dispersed distribution of the mitochondria in the control, whereas they are more perinuclear in the FAD-PS1^M146L^ and FAD-PS1^A246E^ cells. **b** MEFs in which PS1 was knocked down (by small hairpin RNA). Cells were stained as in **a**. Note relatively dispersed distribution of the mitochondria in the control, whereas they are more perinuclear in the PS1-knockdown cells. This phenotype could be rescued by overexpression of WT human PS1, but not by expression of a human pathogenic PS1 mutation (A246E)

**Fig. 2:**
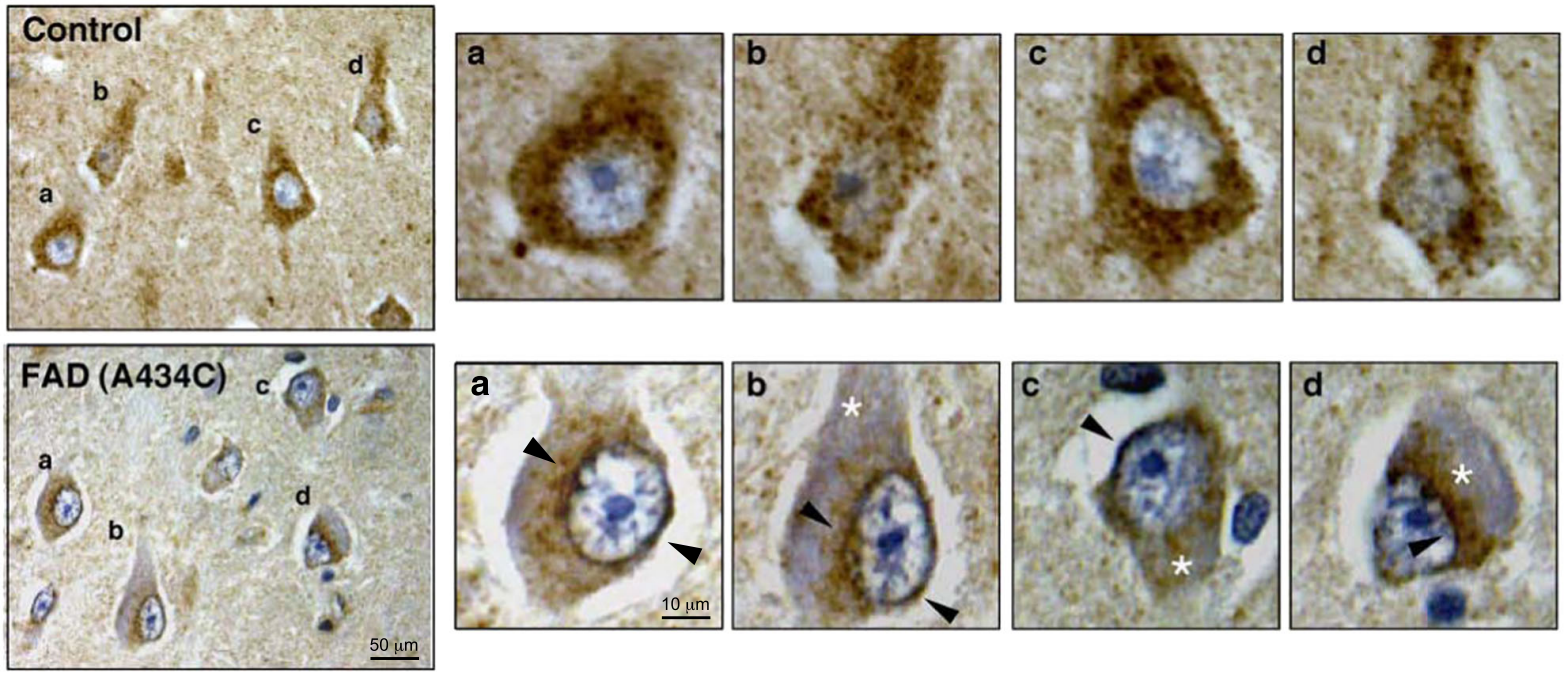
Immunohistochemistry to detect mitochondria (FeS subunit of complex III of the mitochondrial respiratory chain) in the hippocampus (CA1 region) from a FAD patient with a PS1 mutation (A434C). (Upper panels) Control subject (left), with four indicated neurons (**a**–**d**) magnified at right. Note relatively uniform stain (brown), indicating a homogeneous distribution of mitochondria in the cell body. (Lower panels) FAD-PS1^A434C^ patient. Notation as in upper panel. Note the perinuclear distribution of immunostain (brown rings; arrowheads), with a relative paucity of immunostain in the distal regions of the cell body (asterisks)

Besides the effects on bioenergetics, there are also significant changes in mitochondrial dynamics and localization in AD cells, namely, dysfunctional mitochondrial axonal transport^19,47–49^, deregulated organellar dynamics (e.g., mitochondrial fission and fusion)^17,50–53^, and a more perinuclear distribution of the organelles^17,50,54,55^. On this latter point, we too have observed perinuclear mitochondria in AD patient cells (Fig. 1a) and were also able to reproduce the perinuclear phenotype in mouse embryonic fibroblasts (MEFs) in which endogenous PS1 mRNA was knocked down (Fig. 1b). Equally important, we observed a reversal of the perinuclear phenotype upon overexpression of the wild-type allele of human PS1, but not a pathogenic AD-mutant allele (A246E) (Fig. 1b). Consistent with this, we obtained a similar result in the most clinically relevant tissue, namely human brain. Specifically, we used immunohistochemistry to detect mitochondria in the hippocampal CA1 region of an autoptic brain from an AD patient with a pathogenic PS1 mutation (A434C)^56^. Mitochondria were uniformly distributed in the cytosol of control hippocampus, as expected. In contrast, we detected a “ring” of mitochondria around the nucleus of the patient neurons, and depletion of the organelles in the distal region of hippocampal cell bodies (Fig. 2). Thus, we believe that the observation of perinuclear mitochondria in patient cells in vitro likely reflects what is occurring clinically in vivo. Moreover, it appears that γ-secretase activity and/or APP processing play a role in the altered distribution of mitochondria in at least some forms of AD^17,50^, but the relationship, if any, of mitochondrial maldistribution to altered APP processing has been obscure; this issue is addressed below.

Taken together, it is clear that there are numerous functional alterations in mitochondrial behavior in both familial and sporadic AD. However, even though many of these mitochondrial phenotypes are evident before the appearance of plaques^57–59^, we believe that mitochondrial dysfunction likely will not be found to be the underlying cause of the disease, for a number of reasons.

First and foremost, patients with authentic mitochondrial diseases (by which we mean diseases where bioenergetic deficits are the initiating cause of the pathology), whether due to mutations in the mitochondrial or nuclear genomes, do not evince the symptomatology of AD, even in those patients who live into their fourth and fifth decades. Generally speaking, mitochondrial diseases are characterized by numerous defects (e.g., encephalopathy, myopathy, endocrinopathy, retinopathy, and gastrointestinal and kidney disorders) that are simply not seen with any frequency in AD.

Second, mitochondrial deficiency is a common consequence of other insults, such as tissue injury. In fact, the altered mitochondrial function and dynamics seen in AD are also seen in a number of other neurodegenerative disorders that are distinct from AD. For example, perinuclear mitochondria are seen in Huntington disease^60,61^ and in amyotrophic lateral sclerosis^62–65^, and was seen in a patient with a mutation in the mitochondrial fission protein DRP1^66^.

Finally, perinuclear mitochondria can be induced by overexpression of the mitochondrial fission protein FIS1^67^ and also, notably, by tau^68^. Thus, these findings imply minimally that mitochondrial dysfunction and altered mitodynamics, while occurring early, are probably downstream consequences of other specific primary events in AD progression, and are not a fundamental cause of pathogenesis.

### Mitochondria and genetics

Genetically, alterations in mitochondrial DNA (mtDNA) have been found in AD cells and tissues^69^. These include both qualitative changes, such as the association of specific mtDNA haplogroups with the risk of developing AD^70–73^ and the presence of mtDNA deletions^74,75^ and point mutations^76–78^ in patient cells and tissues, and quantitative changes, such as reduced mtDNA levels in AD cerebrospinal fluid^79^. At the gene expression level, one study showed that the transcription of a number of nuclear-encoded oxidative phosphorylation (OxPhos) subunits was reduced in AD blood, whereas transcription of most mtDNA-encoded subunits was elevated in AD blood^80^.

Nevertheless, from the genetic point of view, in spite of the implication that mtDNA mutations, and especially point mutations, should result in maternally inherited AD, only a tiny number of mtDNA variants have been ascribed specifically to the development of the disease^76^, and even this is controversial^81–83^; in fact, there is little evidence for maternal inheritance in AD at all^84,85^.

Finally, genetic association studies have identified numerous nuclear loci associated with increased risk for developing AD^86^. Along with the three FAD-linked genes (*APP*, *PSEN1*, and *PSEN2)*, the most commonly accepted sporadic AD (SAD) risk loci (i.e. linkage to mutations either near or within known genes) are *ABCA7*, *APOE*, *BIN1*, *CASS4*, *CD2AP*, *CD33*, *CELF1*, *CLU*, *CR1*, *EPHA1*, *FERMT2*, *INPP5D*, *MEF2C*, *MS4A*, *NME8*, *PICALM*, *PLD3*, *PTK2B*, *SLC24A4*, *SORL1*, *TREM2*, and *ZCWPW1*. Notably, none of the proteins encoded by these genes are targeted to mitochondria. Of course, this lack of correlation does not prove that mitochondria are not involved in AD pathogenesis, but by the same token it provides little genetic support to the mitochondrial cascade hypothesis.

### Mitochondria and γ-secretase

In support of the mitochondrial cascade hypothesis, there is an intriguing connection between mitochondria and the γ-secretase complex. First, APP and/or Aβ^44,46,87–94^, as well as components of the γ-secretase complex^95–98^, have been reported to be at or in mitochondria, presumably implicating the organelle directly in AD. Second, there are mitochondria-mediated alterations in APP processing in AD cells and tissues^39^. Third, PS1 enhances the expression of PGC-1α, the master regulator of mitochondrial biogenesis, and this effect is reduced in PS1-mutated cells^99^. Finally, incubation of cultured cells and/or isolated mitochondria with Aβ has been shown to have deleterious effects on mitochondrial functions, including effects on respiration^100–104^, protein import^105^, organellar transport^19,47–49^, organellar localization^106^, and organellar dynamics (e.g., mitochondrial fission and fusion)^17,50–53^. These and other reported data reveal an intriguing association between mitochondrial regulation and γ-secretase activity, but whether this link is direct or is mediated by some other, more indirect, mechanism, is not clear.

Regarding the localization studies, it is true that components of the γ-secretase complex can be found on mitochondria, but their relative concentration is very low compared to that in other membranes, such as ER. Moreover, data from our laboratory^107^ and others^108^ showed that presenilins are not imported into mitochondria, implying that while presenilins and γ-secretase exert direct effects on mitochondria, they do not behave as canonical mitochondrial proteins. Another argument in opposition to APP or γ-secretase activity being localized to mitochondria is that this protein complex is only activated in lipid raft domains^109–112^, which do not exist on mitochondrial membranes^113^.

Regarding the toxic effects of Aβ on mitochondria, previous studies showing inhibitory effects of Aβ treatment on mitochondrial function could have been due to the use of unphysiological concentrations of Aβ^101^. Indeed, inhibitory effects on mitochondria were observed with Aβ concentrations that were 10–100 times higher than those found in the entorhinal cortex or cerebrospinal fluid from AD patients^114^.

We therefore think that the pathogenesis of AD cannot be explained as resulting from those alterations in mitochondrial function that are similar to those seen in authentic mitochondrial disorders. Nevertheless, mitochondrial dysfunction is an undeniable early symptom of the disease that needs to be investigated and understood if we are to understand better the course of AD pathogenesis. How might this conundrum be reconciled?

## Mitochondria-associated ER membranes, APP processing, and bioenergetics in AD

Some years ago we hypothesized that the phenotypes seen in AD, including the mitochondrial disturbances, were the downstream consequences of some primary insult in AD arising prior to plaque and tangle formation, and triggered by mutations in PS1, PS2, and APP in the case of FAD, and by unknown causes in the case of SAD^115,116^.

With that in mind, we first tried to clarify the subcellular localization of presenilins and γ-secretase activity and its spatial relationship to mitochondria. Intriguingly, our group found^107^, and others confirmed^117–119^, that presenilins and γ-secretase activity, while localized at the ER, as described previously^120^, are enriched in mitochondria-associated ER membranes or MAM. MAM is a specialized subdomain of the ER that, as opposed to the rest of the ER, has the features of a lipid raft and is rich in cholesterol and sphingomyelin^121,122^. MAM is critical for processes that occur at the interface between mitochondria and ER, including phospholipid biosynthesis, cholesterol esterification, calcium transport, and communication between the two organelles^123^.

After this initial finding, our group (and subsequently others^124–126^) became interested in the potential role that MAM might play in the pathogenesis of AD^115,116,127,128^. We therefore measured MAM activity and ER-mitochondrial connectivity in AD cell models and in cells from AD patients, and found both to be increased significantly compared to controls^122^.

This connection between MAM and AD, at least on theoretical grounds, is appealing, because besides its close apposition to mitochondria, many of MAM’s known functions are among the functions that are perturbed in AD beyond the accumulation of amyloid plaques and tangles^129,130^. These include the regulation of phospholipid, cholesterol, and calcium homeostasis^123^, increased ER stress^131–133^, and perturbed calcium homeostasis, driven, in part, by interactions between p53 and the sarco-ER calcium pump at the MAM^132^. In addition, at least one known MAM-localized enzyme, acyl-CoA:cholesterol acytransferase (ACAT1; gene *SOAT1*)^134^ appears to be required for the production of Aβ^135,136^. Moreover, altered mitochondrial function might somehow be connected to APP processing^137^, at least in the familial form of the disease where APP processing is clearly perturbed^2^. Finally, given the physical proximity of ER to mitochondria^123,134,138^, it is possible that the mitochondrial disturbances that are found in AD might also be due to perturbed MAM morphology and behavior^133,139–141^. For example, given the fact that the subcellular localization of the majority of ER is perinuclear, the increased apposition between ER and mitochondria in AD^122^ could help explain why mitochondria accumulate in the perinuclear region in the disease. In order to address whether perturbations in MAM are responsible for mitochondrial dysfunction in AD, we focused on the relationships among APP processing, MAM behavior, and mitochondrial regulation.

In the non-amyloidogenic pathway, full-length APP (~700 amino acids (aa) in length) is first cleaved by α-secretase at the plasma membrane to produce a long soluble N-terminal fragment (sAPPα) and a short membrane-bound 83-aa C-terminal fragment, called C83; C83 is cleaved by the γ-secretase complex to produce two peptides, P3 and the APP intracellular domain (AICD)^142^. In the alternative amyloidogenic pathway, full-length APP is first cleaved by β-secretase (BACE1) within endosomes to produce a slightly shorter soluble N-terminal fragment (sAPPβ) and a slightly longer 99-aa membrane-bound C-terminal fragment, called C99. C99 is then delivered to the ER, via a currently unknown mechanism, to be cleaved by the γ-secretase complex, producing two peptides, Aβ and AICD. In unaffected individuals, C99 is cleaved rapidly to Aβ_40_, which is ~40 aa in length. In AD, C99 is cleaved to Aβ_42_, which is ~42 aa in length, and there is an increase in the ratio of Aβ_42_:Aβ_40_. Since Aβ has been found to be produced in MAM^107,118^, it was logical to assume that the substrate for MAM-localized γ-secretase, namely C83 and/or C99, must be present in this compartment. In agreement with this supposition, we found that C99 (but not C83) was present not only in endosomes, as expected, but in MAM as well, both in cells and in tissues^143^, where it undergoes cleavage by γ-secretase to generate Aβ^107,117,118^.

Our data further showed that in cellular models of AD, in cells and tissues from AD animal models, and in cells from FAD and SAD patients, there were significant increases in C99 in MAMs that correlated with alterations in MAM structure and function^143^. Specifically, we found that the accumulation of C99 at MAM resulted in the upregulation of sphingomyelin hydrolysis by sphingomyelinases (SMases) within this ER subdomain, but the identity of the specific SMases that are upregulated (there are at least five) is currently unknown. We were also able to replicate the increase in SMase activity at MAM domains in SH-SY5Y cells by inhibiting γ-secretase activity (thereby promoting the accumulation of C99). Supporting this result, the inhibition of BACE1 activity (which reduces C99 formation) resulted in an attenuation of SMase activity^143^.

This increase in SMase activity resulted not only in reductions in the content of sphingomyelin but also in a notable elevation of the sphingomyelin hydrolysis product, ceramide^143^. This finding was noteworthy because ceramide is not only a pro-apoptotic molecule^144^ but is also an inhibitor of mitochondrial respiration^145–149^. Indeed, we found that in our presenilin-mutant and AD patient cells, ceramide levels were elevated and respiratory chain function was decreased, as was respiratory supercomplex formation and function^143^. Importantly, manipulation of the ceramide pathway (both pharmacologically and genetically) to reduce ceramide levels in these cells reversed the bioenergetic defects^143^. In addition, reduction of C99 levels, either via inhibition of BACE1 activity (again, both pharmacologically and genetically) or via ablation of the *APP* gene, also reversed the bioenergetic deficits, concomitant with a renormalization of the sphingolipid profiles^143^. Importantly, these phenotypes could not be replicated by the addition of physiological concentration ratios of Aβ_42_:Aβ_40_, physiological concentrations of Aβ_42_ oligomers, or by the overexpression of AICD. We therefore believe that the bioenergetic defects in AD are likely to be the consequence of upregulated sphingolipid turnover and increased ceramide content triggered by the accumulation of C99 at the MAM. This elevation in ceramide levels alters mitochondrial membrane properties, hindering the assembly and activity of respiratory supercomplexes, resulting or exacerbating, at least in part, in bioenergetic deficiencies. Importantly, these findings implicating C99 are consistent with the findings of others, who showed that altered APP processing, especially via MAM-localized PS2^126,150^, decreased bioenergetics. Interestingly, deletion of a portion of the C99 transmembrane region altered mitochondrial morphology and function in HeLa cells, including decreased ATP levels and decreased membrane potential^151^.

These findings are particularly important because the sphingolipid and mitochondrial phenotypes that we found in PS-mutant cells and in cells from FAD patients were also observed in cells from SAD patients^143^, in which the *PSEN1*, *PSEN2*, and *APP* genes are normal. This latter result implies that from a mitochondrial point of view, both the familial and sporadic forms of the disease have a common pathogenetic origin. In this regard, we note that the most important genetic risk factor for developing SAD is a variant of apolipoprotein E (ApoE), a protein required to ferry cholesterol within lipoprotein particles: the ε4 allele (ApoE4) confers a significantly higher risk of developing AD than does the ε3 allele (ApoE3)^152^. It is noteworthy, therefore, that ER-mitochondrial communication and MAM function were increased significantly in fibroblasts and neurons treated with ApoE4-containing astrocyte-conditioned media as compared to those treated with ApoE3-containing astrocyte-conditioned media^153^. Moreover, in spite of no obvious qualitative defect in the *APP* or presenilin genes in SAD, C99 is nevertheless elevated in these patients^154–156^.

In summary, previous data and our own results point to a direct connection between APP processing and OxPhos deficiency via C99, both in FAD and SAD. Moreover, these data imply that at least from the mitochondrial point of view, it is C99, and not Aβ (nor any other APP processing product (e.g., sAPPα, C83, or P3)^137^), that is the key APP-processing intermediate that is required for pathogenicity.

## Concluding remarks—the “MAM hypothesis”

We believe that while mitochondrial dysfunction is an early and significant defect in AD, it is not a primary insult in the pathogenesis of the disease, but rather is a consequence of MAM dysfunction that is driven by an increased presence of C99 at MAM.

**Fig. 3:**
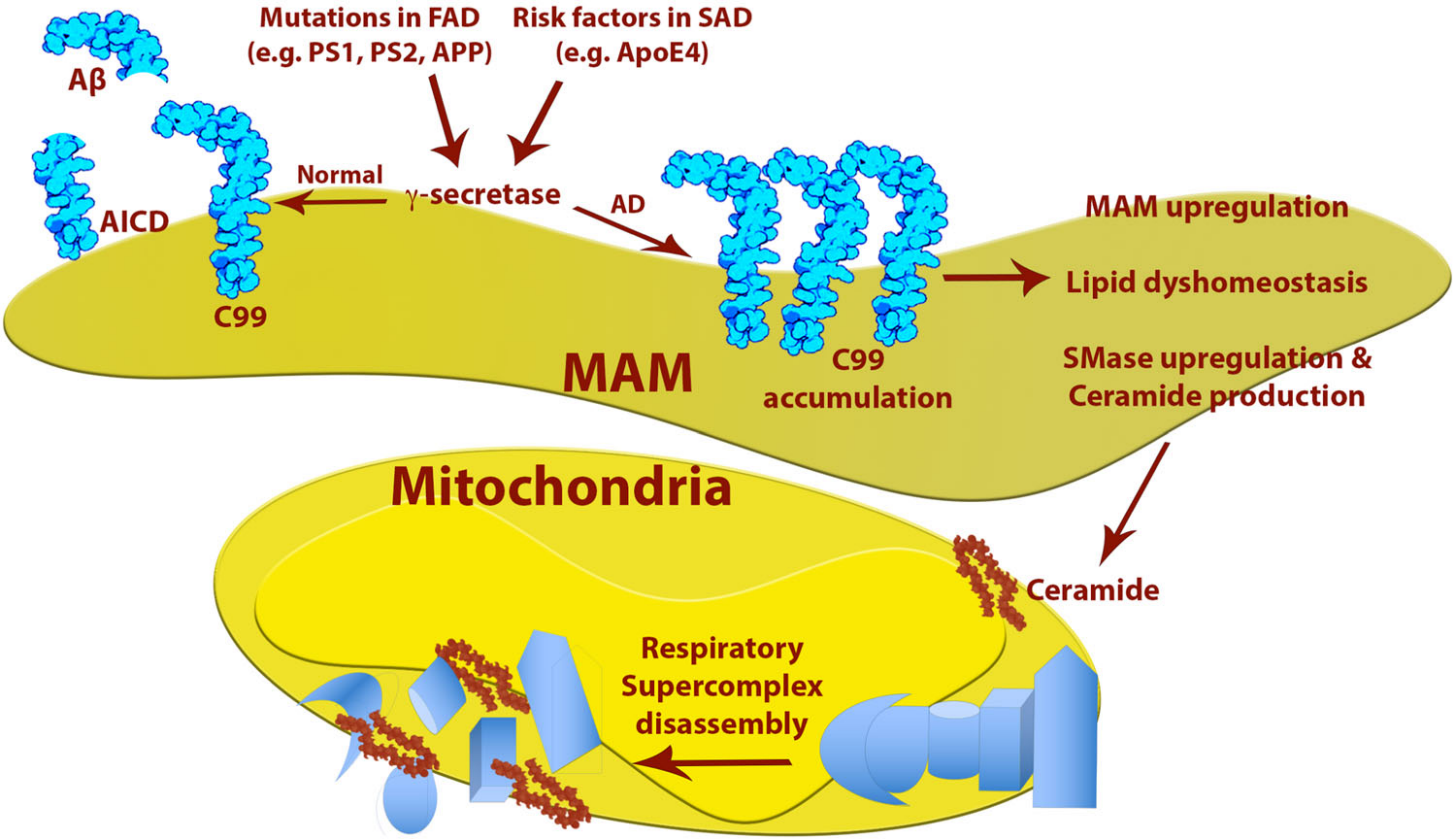
Model of the “MAM hypothesis” for the pathogenesis of AD, with emphasis on the mitochondrial alterations. See text for details

These results also imply that from the genetic standpoint, dominant mutations in PS1 likely result in haploinsufficiency^157–159^ or behave in a dominant-negative manner^160^ rather than resulting in a gain of function^161^, with the reduced PS1 activity as the likely cause of the increased levels of C99. In turn, increased MAM-localized C99 promotes the various features of the disease, including the calcium and lipid dyshomeostasis, the mitochondrial perturbations, and ultimately the plaque and tangle formation. In agreement with this view, the accumulation of C99 in mitochondrial fractions, and mitochondrial respiratory chain deficiency, has been detected in brains from animal models of AD^162^, and was reversed following deletion of *BACE1* (thereby preventing the formation of C99)^162^. Finally, the idea that mitochondrial dysfunction is the result of the accumulation of C99 in the MAM rather than being a consequence of higher levels of Aβ_42_ helps explain previous results showing that mitochondrial alterations occur early in the pathogenesis of the disease^58^, well before any pathophysiological hallmark of AD becomes apparent^57^. Nevertheless, we believe that even though mitochondrial dysfunction is an early event upstream of plaque and tangle formation, we do not consider the organelle to be a reasonable target for therapeutic intervention, as the mitochondrial perturbations observed in AD are themselves consequences of an even earlier precipitating process, namely elevated C99 and altered lipid homeostasis (Fig. 3). Thus, it is possible that increased ER-mitochondrial connectivity and upregulated MAM behavior underlie the metabolic disturbances (and probably the other phenotypes) seen in AD^122,143,163^ (the “MAM hypothesis”^115,116,127,128^). We are currently actively engaged in deducing the mechanism(s) underlying these changes.

Finally, alterations in ER-mitochondrial communication and in MAM behavior may not be confined to AD. Other neurodegenerative disorders, such as Parkinson disease and amyotrophic lateral sclerosis, also evince altered mitochondrial function and disturbances in calcium and lipid homeostasis^164^. Notably, alterations in MAM behavior have also been found in both of these disorders, and are especially prominent in the familial form of these diseases, where a connection between the culprit gene and altered MAM behavior can be drawn^165,166^. Thus, altered ER-mitochondrial communication has the potential to play a critical, and hitherto unappreciated, role in the pathogenesis of many of the most common and devastating diseases of advanced age^129^.
